# Efficacy of Osimertinib in Lung Squamous Cell Carcinoma Patients with *EGFR* Gene Mutation–Case Report and a Literature Review

**DOI:** 10.3390/curroncol29050285

**Published:** 2022-05-13

**Authors:** Anna Rekowska, Piotr Rola, Magdalena Wójcik-Superczyńska, Izabela Chmielewska, Paweł Krawczyk, Janusz Milanowski

**Affiliations:** 1 Student Scientific Association, Department of Pneumonology, Oncology and Allergology, Medical University of Lublin, 20-059 Lublin, Poland; piotr99rola@gmail.com; 2 Department of Pneumonology, Oncology and Allergology, Medical University of Lublin, 20-059 Lublin, Poland; magdalena.wojcik-superczynska@umlub.pl (M.W.-S.); izabela.chmielewska@umlub.pl (I.C.); pawel.krawczyk@umlub.pl (P.K.); janusz.milanowski@umlub.pl (J.M.)

**Keywords:** osimertinib, squamous cell carcinoma, non-small cell lung cancer, EGFR, TKIs

## Abstract

Non-small cell lung cancer (NSCLC) is the most common type of lung cancer and the leading cause of cancer-related mortality worldwide. It is responsible for 80–85% of lung cancer cases. NSCLC can be divided into several groups, led by adenocarcinoma (ADC)–40–50% and squamous cell carcinoma (SCC)–20–30%. The development of new molecular therapies targeting particular abnormalities such as mutations in the *EGFR* (*Epidermal Growth Factor Receptor*) gene or *ROS1* or *ALK* genes rearrangements resolved in novel strategies in advanced NSCLC management. *EGFR* mutation occurs mostly in patients with ADC and those patients are mostly females with no or light smoking history. The hereby presented patient fitted the ADC characteristics, while they were diagnosed with SCC. The unusual diagnosis implied further genetic testing, which established the occurrence of L858R substitution in exon 21 in the *EGFR* gene. A 63-year-old female was admitted to the unit due to a dry cough, pain in the right chest area and dyspnoea. When diagnosed, the patient had a peripheral mass in the right lung superior lobe (55 × 40 mm), satellite nodules in the apex of the same lung and packets of disintegrating lymph nodes. Positron Emission Tomography (PET-CT) confirmed a diffuse neoplastic process qualified as stage IV on the TNM scale. Due to *EGFR* gene mutation, the woman was administered osimertinib, however, the treatment did not succeed, and other therapeutic solutions were undertaken. The patient died 10 months after diagnosis. Patients with advanced ADC harboring *EGFR* mutation can receive osimertinib, a third-generation tyrosine kinase inhibitor (TKI), however, the use of TKIs in SCC remains controversial. In some published cases, osimertinib treatment led to success, in others, the therapy did not result in the expected final effect. Small sample groups and diverse molecular backgrounds indicate the need for further research in this field. Thus, the treatment decision-making process in those patients overall remains extremely demanding and ambiguous.

## 1. Introduction

Non-small cell lung cancer (NSCLC) accounts for about 80–85% of all lung cancer cases and lung cancer is a major cancer-related cause of death worldwide [1]. Adenocarcinoma (ADC) is the most common NSCLC subtype with approximately 40–45% of cases, followed by squamous cell carcinoma (SCC) with 20–30% of cases [2,3]. Besides standard platinum-based chemotherapy, NSCLC patients have access to new promising therapeutic options. Molecularly targeted therapies could be used in advanced non-squamous NSCLC patients with mutations in the *EGFR* (*Epidermal Growth Factor Receptor*) gene or with *ROS1* or *ALK* gene rearrangements. *EGFR* is considered to be one of the most frequent driver genes in NSCLC [4]. After excluding the presence of genetic abnormalities, it is possible to use chemoimmunotherapy or immunotherapy, according to the PD-L1 (Programmed Death-Ligand 1) expression status on tumor cells. SCC patients rarely harbor *EGFR* mutations and, therefore, the use of EGFR tyrosine kinase inhibitors (TKIs) remains controversial in such patients. Advanced SCC patients are usually treated with immunotherapy or chemoimmunotherapy. Currently, neither guidelines nor clinical trial results carry official data that could unanimously confirm or deny the legitimacy and efficiency of EGFR TKIs in patients with *EGFR* positive SCC. However, first attempts have already been made with various outcomes and the procedure needs further trials to determine its possible usefulness in SCC treatment. Since the available data do not yet allow us to make certain and universal therapeutic guidelines regarding EGFR mutated in SCC patients, in this case, the treatment decision-making process overall remains extremely demanding and ambiguous.

## 2. Case Presentation

A 63-year-old female patient with no smoking history manifested the following symptoms: dry cough, pain in the right chest area and dyspnoea that suggested conducting a further examination. The radiograph revealed a nodular shadow in the right lung hilum, whereas computed tomography (CT) showed a mass in the perihiliar area of the right lung superior lobe in dimensions of 55 × 40 mm. Moreover, two satellite nodules (27 × 21 mm and 34 × 33 mm) in the right lung apex and packets of disintegrating lymph nodes were also shown. Endobronchial ultrasound with transbronchial needle aspiration (EBUS-TBNA) was performed. Moreover, the patient underwent thoracoscopy to assess the lymph node metastases. Patomorfological examination of both materials led to the identification of poorly-differentiated SCC. However, an atypical profile of the SCC patient (young, female and non-smoking) was an indication for a genetic test, that revealed L858R substitution in exon 21 in the *EGFR* gene. *EGFR* gene mutation testing by the real-time PCR technique was performed twice using EBUS-TBNA and thoracoscopy materials. According to the National Drug Program, harboring *EGFR* mutation excludes NSCLC patients from further molecular testing. Therefore, the status of PD-L1 remains unknown and the patient could not receive immunotherapy. Additionally, at the time of the diagnosis, chemoimmunotherapy lacked reimbursement and the woman could not receive it in first-line treatment either. However, considering the low immunogenicity of NSCLC harboring oncogenic driver mutation and therefore poor responsiveness to the immunotherapy, the potential benefit of its use in our patients is doubtful [5,6]. The patient also underwent the Positron Emission Tomography (PET-CT), which confirmed the diagnosis of the diffuse neoplastic process (stage IV on the TNM scale).

**Figure 1:**
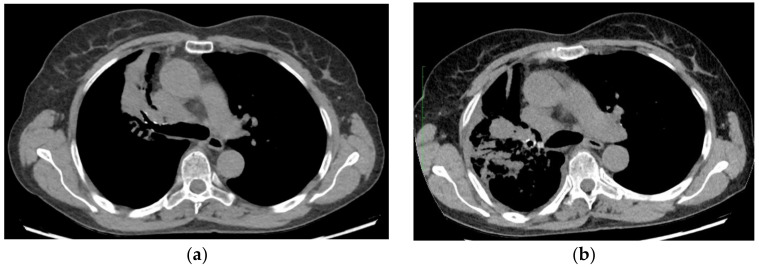
Computed tomography (CT) images of the lung (**a**) CT scan 1 month after first osimertinib administration (**b**) CT scan 3 months after first osimertinib administration.

The patient, because of the presence of *EGFR* mutation, received non-standard first-line treatment with the third generation of EGFR TKI. The woman signed the consent for the proposed treatment and the consent was included in the patient’s documentation. Osimertinib was administered within an emergency drug access procedure. During the EGFR TKI therapy, the patient’s general condition and quality of life remained stable (performance status 1 according to ECOG/WHO criteria). Nevertheless, CT revealed significant progression of the disease after three months of treatment. The tumor mass did not decrease in size and the cancer had metastasized. Three months into the administration enlargement of mass in the superior lobe with the addition of metastatic nodular changes, atelectasis, and respiratory volume reduction were observed (Figure 1). Other new CT findings such as nodules in dimensions of 6 mm were also found in the apex of the left lung and left segment 6. A mass in the right hilum, that currently measured 50 × 38 × 58 mm and infiltrated the mediastinum, was causing a narrowing of the superior vena cava. Additionally, CT revealed metastatic nodular changes of the isthmus and thyroid lobes, liver, and left adrenal gland adjoining the left renal vein. Neoplastic lesions in both kidneys and osteolytic lesions in multiple vertebrae, left pelvic bone and neck of the left femur were also presented. The weakness of our article is the fact that the patient did not have next-generation-sequencing (NGS) testing to detect the reason for primary drug resistance.

Due to further progression of the neoplastic process, almost 4 months after the first osimertinib administration, the patient was qualified for the second-line treatment. The woman did not consent to have another biopsy. However, the fact, that the patient progressed within such a short period of time speaks for the tumor’s primary resistance to the TKIs and not the acquired one. Additionally, at that point reattempting molecular diagnostics was not crucial considering the fact that the woman received second-line therapy consisting of chemotherapy (cisplatin and vinorelbine). She tolerated the first cycle well. However, before the second cycle, breakthrough pain and deep vein thrombosis occurred. The third cycle was preceded by the development of central pulmonary embolism. Nevertheless, liver metastatic and adrenal changes were in partial remission. Slight regression was also noticed in the right hilum. Soon after, the dose of cytostatics was reduced by 25% due to anemia. After the fourth cycle of chemotherapy, progression of the disease and bone marrow insufficiency were revealed. The chemotherapy was stopped. The patient underwent embolization of metastatic liver tumor, stereotactic radiotherapy for metastatic changes and palliative radiotherapy for the mass in the right lung. Within the four months of osimertinib treatment, the patient did not benefit from it and the clinical status worsened. Chemotherapy as a second-line treatment led to slight temporal improvement. Nevertheless, adverse effects exceeded potential benefits and systemic treatment was ended. She died ten months after receiving the diagnosis.

## 3. Discussion

Adenocarcinoma and squamous cell carcinoma are both subtypes of non-small cell lung cancer. However, those two types of NSCLC differ not only in terms of cell morphology but also in localization, therapeutic options, prognosis and patient characteristics. Studies strongly associate tobacco use and male sex with SCC, meanwhile, ADC is predominant in non-smoking or light smoking females [7]. *EGFR* mutations can be observed in around 8.5–15% of Caucasians and in 40% of Asian patients with adenocarcinoma [8,9,10,11]. Only 1–1.5% of SCC patients harbor the *EGFR* mutations, and therefore, due to the low frequency of occurrence, these mutations are not tested routinely [12,13,14].

EGFR is a transmembrane protein and, after binding the ligand, leads to a cascade of processes resulting in increased DNA replication, stimulation of cell division, angiogenesis and blockade of apoptosis [15]. Approximately 85–90% of NSCLC patients with *EGFR* mutations presented deletion in exon 19 and L858R substitution in exon 21 and those indicate the legitimacy of EGFR TKIs use [16]. Gefitinib and erlotinib belong to first-generation EGFR TKIs, afatinib and dacomitinib–to second-generation EGFR TKIs and Osimertinib–to the third generation of EGFR TKIs. Osimertinib is also effective in patients resistant to previous EGFR TKIs generations with T790M mutation in exon 20 of the *EGFR* gene and in cases where first-generation TKIs did not succeed, with a median progression-free survival (PFS) of 10.1 months, which is 5.7 months longer comparing to platinum-based therapy plus permetrexed [17,18]. The examination of *EGFR* mutation in SCC patients is reasonable and suggested mainly when the patient’s profile does not match the typical phenotype of SCC, such as female and non-smokers. Among *EGFR* mutation-positive cases, about 75% were female and more than 80% had never smoked [1]. Based on the fact that women account for only 10–15% of SCC cases and only a small part of them have never smoked, the potential testing group is very limited [19]. The amount of studies establishing the rates of occurrence of *EGFR* mutations in SCC patients is limited. However, according to available data, the percentage of female SCC patients harboring *EGFR* mutation ranges from 14 to 33%. Moreover, in this *EGFR*-mutated group, 93.5 to 100% never smoked [10,20,21,22]. Female sex as the main criteria for *EGFR* mutation detection not only allows us to significantly reduce the potential testing group but also ensures detection of a considerable percentage of the patients harboring *EGFR* mutation who could potentially receive and benefit from the TKIs use.

Thus, in certain groups of SCC patients, the probability of *EGFR* mutation occurrence increases, and both the National Comprehensive Cancer Network (NCCN) and the European Society for Medical Oncology (ESMO) recommend *EGFR* testing in SCC According to the guidelines, not only among non-smoking females but in all non-smokers (NCCN, ESMO) and long-time ex-smokers or light-smokers (<15 pack-years) (ESMO) [23,24].

The last decade brought progress in NSCLC treatment. The 2-year survival rate increased from 34% in 2009–2010 to 42% in 2015–2016 and is continuously growing [25]. The breakthrough was possible i.a., thanks to the introduction of targeted therapies, including *EGFR, ALK* and *ROS1* inhibitors that are more efficient than traditional chemotherapy [15]. However, to apply one of them, it is necessary to detect and identify genetic alterations that condition the legitimacy and predictive responsiveness of the therapies. Most of the genetic alterations occur in non-squamous NSCLC and treatment possibilities for SCC patients are limited. It results in decreased 5-year survival rates, in SCC compared to ADC patients: for stage III–13% vs. 27% and for stage IV–2% vs. 6%. Previous studies revealed that *EGFR* mutated SCC patients treated with EGFR TKIs showed shorter PFS and overall survival (OS), however, observation biases are possible due to the small sample of the groups. According to Jin R, PFS in SCC patients presented with several *EGFR* mutation types, treated with TKIs did not vary [26]. Gender or smoking history had no impact on PFS in the study population either. Two mutations mentioned as the most frequent activating ones in the EGFR gene are exon 19 deletion and exon 21 Leu858Arg mutation. As claimed by the Japanese Joint Committee of Lung Cancer Registry, OS in non-ADC patients with exon 19 deletion is longer than in those with L858R mutation [27,28].

Besides *EGFR*, another significant and even more frequent driving mutation in NSCLC is the Kirsten rat sarcoma virus oncogene homolog (*KRAS*) present in 20–25% of all NSCLC cases [29]. Recently, significant advances in developing *KRAS* targeted therapies were made, even though *KRAS* was considered to be “undruggable” for the past decades. Two *KRAS G12C* inhibitors- sotorasib and adagrasib obtained promising results in clinical trials. However, the occurrence of *KRAS* mutation is almost exclusive to tobacco users and ADC patients. The prevalence of *KRAS* mutations in ADC reaches up to 25% in the Caucasian population and 10% in Asian patients. In SCC *KRAS*, mutation is quasi non-existing [29,30,31,32]. Therefore, the potential detection of KRAS mutation and the use of targeted therapies may not be fully applicable in the case of non-smoking SCC patients.

The first attempts to treat SCC harboring *EGFR* mutation with the third generation of EGFR TKI have already been made and published, inter alia, by researchers from the University Hospital of Udine. Authors reported SCC 54-year-old male patient with metastatic SCC and occasional smoking history. Genetic analysis revealed inframe deletion in exon 19 of the *EGFR* gene that coexisted with T790M mutation in exon 20 and G724S mutation in exon 18 of the *EGFR* gene, as well as P152L mutation in *TP53* gene. The administration of two cycles of cisplatin and gemcitabine ended with progression. Therefore, erlotinib treatment has been undertaken. Six months from the start of treatment, a CT scan revealed further progression of the disease. Liquid biopsy confirmed the presence of T790M mutation in the *EGFR* gene. Thus, osimertinib was administered with no apparent benefit to treatment. Progression led to a nivolumab treatment attempt, and due to the ineffectiveness of this therapy, the decision to return to erlotinib was made. All these activities proved ineffective [33].

A cohort study performed in the Zhejiang Province investigated genetic alterations in three groups of stage IV SCC patients–*EGFR*-mutant lung SCC, *EGFR*-mutant lung ADC and wild-type lung SCC between June 2015 and June 2019. According to patients’ genomic profiles published in The Cancer Genome Atlas (TCGA) and other Chinese cohort studies, the most frequent genetic abnormalities occurring in SCC and ADC patients differ. Among SCC patients, *TP53, NFE2L2, CDKN2A, KEAP1* and *PTEN* gene mutations were observed mostly and in ADC patients *TP53*, *KRAS*, *EGFR*, *STK11* and *RB1* genes were the most frequent ones. The undergone study presented a higher occurrence rate of NF1, ATR and BRCA1 in SCC patients than ADC ones, from a sample group, possible negative impact on PFS co-occurrence of *EGFR* mutation with either *CREBBP, ZNF217* or Wnt pathway mutation while *GRM8* could be associated with longer PFS. However, even despite complex research, focused on the genomic investigation of the poor and heterogeneous responsiveness of *EGFR* mutated SCC to TKIs, the background of this phenomenon could not be fully and precisely established [26].

Fassunke J., et al., demonstrated that G724S mutation in the *EGFR* gene limits the activity of the third generation of EGFR TKI in both in vitro models and in vivo observations. Structural analyses and computational modeling indicated that this mutation may induce a conformation of the glycine-rich loop, which is incompatible with the binding of third-generation TKIs [34].

Peng MY et al., described a 50-year-old male Asian patient with locally advanced SCC and *EGFR* exon 19 deletion. Due to the resistance to the first and second generation of EGFR TKIs, resulting from the occurrence of the T790M mutation, the patient received osimertinib. Response to the treatment was observed. Lobectomy confirmed pathomorphological complete remission of the primary lesion and metastatic lymph nodes. The patient obtained full recovery with no signs of recurrence in 8 months follow-up [35].

Another two cases were patients of the Niigata University Graduate School of Medical and Dental Sciences. The first case was a 67-year-old female SCC patient who reported 23 pack-years of smoking and was diagnosed in the IIIB stage. The patient received chemoradiotherapy with partial response. Due to the recurrence of the primary lesion and metastases to the central nervous system, she underwent radiotherapy. Subsequently, the patient received osimertinib in a standard dose because the tumor cells harbored an exon 19 deletion in the *EGFR* gene. Two months after initial administration, the primary lesion and metastases showed partial remission. Moreover, the patient developed osimertinib-induced pneumonitis that was successfully treated with prednisolone. The second case concerned a 63-year-old male SCC patient, who reported a 45 pack-years of smoking history and was diagnosed in IVB stage. The patient had L858R substitution in the *EGFR* gene. He manifested a right lung tumor, bilateral involvement of the mediastinal lymph nodes and bone metastases that have undergone partial remission by the use of osimertinib in the standard dose [36].

The Japanese Society of Internal Medicine described an SCC patient in the IVA stage with L858R mutation in the *EGFR* gene. A 60-year-old woman with no smoking history had the right lower lobe tumor and metastatic lesions in bilateral lymph nodes and pleura. The patient received afatinib as the first line of treatment with partial response, followed by three failures in further treatment lines consisting of various chemotherapies and immune checkpoint inhibitors therapy. Liquid biopsy, performed after failure of previous therapeutic attempts, detected the occurrence of secondary T790M mutation in the *EGFR* gene. Osimertinib was administered as the fifth line of treatment and resulted in a decrease in tumor size, corresponding to disease stabilization. However, during a 7-month follow-up, an increase in tumor size was observed. Treatment with osimertinib was continued for two more months. The ultimate chemotherapy did not succeed, and the patient died three years after the diagnosis [37].

The Shanghai Chest Hospital data collected between January 2013 and December 2018 includes the medical history of 46 SCC patients with detected *EGFR* mutation. A total of 38 of them received TKIs as the first or second-line therapy, one did not undertake therapy. A total of 36 patients were treated with first-generation TKIs and two with second-generation TKIs. PFS median compared with the group receiving chemotherapy was in favor of patients receiving TKIs (8 vs. 3.2 months) as well as OS median (38.6 vs. 24.9 months). Among 37 patients that progressed, 8 received osimertinib and 2 of them were distinguished with T790M mutation whereas 12 switched to chemotherapy. The OS of both groups reached 30.0 and 21.7 months, respectively. SCC patients harboring *EGFR* mutations benefited from TKI therapy, although compared to the non-mutant EGFR group both reached similar OS median (22,8 vs. 18.6, no statistical difference). According to the study, despite the presence of *EGFR* mutations, TKIs are more effective in ADC than in SCC. Additionally, among the studied patients, the outcomes of the treatment were better in women than in men [20].

Retrospectively, the observational study investigating metastatic lung SCC cases from the Prince of Wales Hospital in Hong Kong between 2000 and 2011 pointed out the possibility of misdiagnosing another subtype–such as adenosquamous carcinoma as SCC, especially when the biopsy sample is small or cells are just mimicking SCC. Statistically, about 5% of all resected NSCLC are adenosquamous carcinoma [38]. Out of four included in the study group patients, one later was reclassified as adenosquamous carcinoma, two harbored L858R activating mutation (females) and one had exon 19 deletion (male). All three of them had no smoking history. A male aged 42 presented with distant metastases and underwent initial lung resection followed by oral erlotinib. Although administration caused a decrease in primary mass and lymph node metastases, new tumors in the lung and liver occurred and the patient died 5 months later. The second patient, a 52-year-old female presented with multiple masses including both lungs, rib, and iliac crest metastases and received gefitinib which led to disease stabilization for a year. However, the patient progressed and seven months later died. The last woman due to localized disease underwent only surgery and radiotherapy. The woman survived [39].

The accuracy of the cytological diagnosis between the subtypes affects the treatment outcomes. This could vary depending on how well-differentiated the cells are or how big the sample was. If adenosquamous carcinoma is misdiagnosed as SCC, because the sample contains only one component, the efficacy of TKI treatment may vary and depend on the composition of the particular tumor [40,41,42]. However, it could also depend on intra-tumor heterogeneity. The mutation may be present in only some of the tumor cells. SCC is considered to have potentially more intratumoral heterogeneity than for instance ADC [43,44,45]. If a sample is collected from more than one region or a new sample is taken while undergoing another diagnostic procedure, the chances of complex and accurate genetic material and therefore proper diagnosis, improve. Therefore, while diagnosing or analyzing study results, this possibility should also be taken into consideration.

## 4. Conclusions

Even though our patient showed no response to osimertinib therapy, some of the described SCC patients with *EGFR* gene mutations benefited from therapy with EGFR TKIs. Previously, the SCC patients could not be included in clinical studies investigating EGFR TKIs efficacy because the *EGFR* mutation in this group is rare and gene testing among them was not as routinely performed, as in ADC. Therefore, the actual data collected from previous studies is limited and clear statements on whether SCC patients benefit from third-generation TKI treatment are still not fully accurate. In the light of small study groups and relatively new therapeutic guidelines, further research is pivotal to determine whether *EGFR* mutated SCC patients could benefit from TKIs just as the ADC ones. By now, only one retrospective study analyzing a larger group of *EGFR*-mutant SCC patients was conducted, however, none of them received third-generation TKIs.

To draw conclusions deprived of biases, prospective studies involving multiple centers and allowing the collection of a sufficient amount of data must be conducted. To provide potential therapeutic guidelines, statistically significant results are indispensable. The investigation of the osimertinib use in *EGFR*-mutated SCC patients should be continued, however, until sufficient reports are established, the treatment should be considered as a demanding and unique process. The need for a personalized and inquiring approach is pivotal as well. Various genetic profiles, sample size, or tumor heterogeneity can affect the course of treatment and outcomes. By now, due to the limited number of SCC patients with *EGFR* gene mutation treated with EGFR TKIs, potential biases should also be taken into consideration in the interpretation of the study results.

## Data Availability

Not applicable.
